# Preclinical development of [^18^F]TAAR1-2203 as a PET radioligand for imaging TAAR1 expression and receptor occupancy

**DOI:** 10.1007/s00259-025-07564-w

**Published:** 2025-11-06

**Authors:** Achi Haider, Zhiwei Xiao, Jiahui Chen, Stefanie K. Pfister, Xin Zhou, Yinlong Li, Ahmad Chaudhary, Chunyu Zhao, Jian Rong, Axel Paehler, Susanne Mohr, Roger D. Norcross, Michael Honer, Linjing Mu, Luca Gobbi, Roger Schibli, Marius C. Hoener, Steven H. Liang

**Affiliations:** 1 Department of Radiology, Division of Nuclear Medicine and Molecular Imaging Massachusetts General Hospital, Harvard Medical School, 55 Fruit Street, 02114 Boston, MA USA; 2 Pharma Research and Early Development, Roche Innovation Center Basel, F. Hoffmann-La Roche, 4070 Basel, Switzerland; 3 Department of Radiology and Imaging Sciences, Emory University, 1364 Clifton Road, 30322 Atlanta, GA USA; 4 Department of Nuclear Medicine, Zhongnan Hospital of Wuhan University, Wuhan, China; 5 Center for Radiopharmaceutical Sciences ETH-PSI-USZ, Institute of Pharmaceutical Sciences ETH, Vladimir-Prelog-Weg 4, 8093 Zurich, Switzerland

**Keywords:** Trace amine-associated receptor 1 (TAAR1), Positron emission tomography (PET), Receptor occupancy, [^18^F]TAAR1-2203, Pancreatic imaging and beta-cell function

## Abstract

**Purpose:**

Trace amine-associated receptor 1 (TAAR1) is an emerging therapeutic target with various implications in neuropsychiatric and metabolic disorders. However, the absence of a suitable positron emission tomography (PET) radioligand has precluded non-invasive quantification of TAAR1 expression and drug-receptor interactions in vivo. In this study, we report the preclinical development of [^18^F]TAAR1-2203, a fluorine-18 labeled PET ligand suitable to image TAAR1 expression in peripheral tissues.

**Methods:**

[^18^F]TAAR1-2203 ([^18^F]RO5263397) was obtained via copper-mediated radiofluorination. In vitro stability was assessed in formulation, serum and using liver microsomes. Specific binding was evaluated by in vitro autoradiography, ex vivo biodistribution, and PET imaging under baseline and blockade conditions with structurally distinct TAAR1 agonists, as well as in TAAR1 knockout (KO) mice. Receptor occupancy studies were conducted in the pancreas – a peripheral organ with high physiological TAAR1 expression.

**Results:**

TAAR1-2203 exhibited high TAAR1 affinity in competition binding assays across species, with K_i_ values of 0.9 nM (mouse) and 5.7 nM (human). Tracer stability was corroborated by cross-species microsomal incubations and ex vivo radiometabolite analyses in mice. In peripheral tissues with known TAAR1 expression, [^18^F]TAAR1-2203 displayed robust signal intensity that was significantly reduced under pharmacological blockade or in TAAR1 KO mice, thus confirming specific receptor binding. Although [^18^F]TAAR1-2203 appeared to cross the blood-brain barrier based on brain time-activity curves and ex vivo radiometabolite analysis, no specific binding was observed in the CNS. Target occupancy studies in the pancreas demonstrated dose-dependent blockade, with a calculated D_50_ of 0.67 µmol/kg for a structurally distinct potent TAAR1 agonist.

**Conclusion:**

[^18^F]TAAR1-2203 represents the first PET radioligand for TAAR1 imaging with favorable in vitro and in vivo performance characteristics, enabling non-invasive assessment of receptor expression and drug occupancy in peripheral TAAR1-expressing tissues. [^18^F]TAAR1-2203 holds promise for translational application in various drug development programs.

**Supplementary Information:**

The online version contains supplementary material available at 10.1007/s00259-025-07564-w.

## Introduction

Trace amine-associated receptor 1 (TAAR1) orchestrates signal transduction through a wide array of agonists, encompassing endogenous trace amines (TA), common biogenic amines, thyronamines, as well as exogenous psychostimulant drugs of the amphetamine class [1]. Notably, trace amines and amphetamines exhibit a greater selectivity for TAAR1 compared to other agonists [1]. Upon activation, TAAR1 signals via Gs proteins to elevate intracellular cAMP levels in response to TAs​ [2, 3]. A strong inverse association between TAAR1 and monoaminergic transporters, particularly the dopamine transporter (DAT), has been established [4, 5]. As such, TAAR1 agonists have been shown to modulate the firing activity of dopamine (DA) and serotonin (5-HT) neurons, eliciting anxiolytic- and antipsychotic-like effects. TAAR1 agonists are currently under clinical evaluation for the treatment of schizophrenia, anxiety disorders, major depressive disorder (MDD), and drug addiction​ [6–12]. Conversely, TAAR1 knock-out animals exhibited an attenuated degeneration of dopaminergic neurons following intrastriatal administration of 6-hydroxydopamine (6-OHDA) in a mouse model of Parkinsonism [13, 14]. Along this line of reasoning, TAAR1 antagonists have been proposed for the management of Parkinson’s disease (PD), however, suitable antagonists with favorable in vivo performance characteristics remain unavailable to date [15].

TAAR1 is expressed in major monoaminergic regions of the mammalian brain, including the ventral tegmental area (VTA), dorsal raphe, striatum, amygdala, hypothalamus and frontal cortex​ [16, 17]. Nonetheless, it remains unclear whether the expression levels in the brain are sufficient to enable targeted positron emission tomography (PET) imaging. Apart from the expression in the brain, the receptor is also expressed in a number of peripheral tissues, including the pancreas, kidney, liver, spleen and gastrointestinal tract [18]. Of particular interest, TAAR1 activation in pancreatic tissue has been shown to enhance insulin secretion in response to glucose challenge [19]. While the precise molecular mechanisms by which TAAR1 promotes insulin secretion from β-cells remain to be fully elucidated, positive modulation of TAAR1 has been linked to beneficial anti-diabetic signaling pathways, suggesting a potential application of TAAR1 agonists as anti-diabetic agents [20]. Additionally, TAAR1 signaling has been implicated in the regulation of gut motility, satiety, and eating behaviors [19, 21], indicating that TAAR1 may play a broader role in metabolic regulation and the pathophysiology of obesity.

Given these broad implications, extensive medicinal chemistry efforts have been devoted to the discovery of potent and selective TAAR1 agonists at F. Hoffmann-La Roche Ltd., resulting in the identification of several exemplary compounds reported previously [8, 22–24]. Among these, structures such as RO5166017, RO5256390, and RO5263397 have demonstrated the ability to prevent psychostimulant-induced hyperlocomotion and stress-induced hyperthermia [8, 24], while RO5203648 exhibited antipsychotic- and antidepressant-like activities [23]. Similarly, TAAR1 modulation was shown to block compulsive, binge-like eating behavior in rats [21]. In line with these observations, TAAR1 agonists have been reported to suppress self-stimulation and compulsive behaviors triggered by substances such as cocaine, methamphetamine (METH), and nicotine [23, 25–31]. Notably, Ulotaront (SEP-363856), a TAAR1 agonist currently in Phase 3 clinical development, has demonstrated efficacy in improving symptoms of schizophrenia in a large randomized, double-blind, placebo-controlled clinical trial​ [32, 33]. These findings collectively underscore the versatile therapeutic potential of TAAR1 agonists across a range of neuropsychiatric and metabolic indications.

Notwithstanding the encouraging advances in TAAR1-targeted drug development, the availability of a suitable imaging tool to visualize TAAR1 and its interactions with potential drug candidates by means of target occupancy studies remains lacking, constituting an unmet medical need. To date, only one TAAR1-targeted PET tracer, [^11^C]TAAR1-1911, has been reported [34]. However, in vivo evaluation of [^11^C]TAAR1-1911 revealed low brain uptake, pronounced metabolic instability, and the presence of brain-penetrant radiometabolites​ [34]. The development of a suitable TAAR1 PET radioligand for the assessment of receptor function in humans would not only facilitate drug development but also potentially serve as a disease biomarker. Of note, developing PET radioligands for central TAAR1 imaging poses a challenge due to the limited abundance of TAAR1 in the healthy mouse brain [12, 35]. While TAAR1 related drug development has traditionally focused on neurological diseases, considerable peripheral expression, for instance in the pancreas, has previously been reported [36]. In this study, we focused on the development of [^18^F]TAAR1-2203 ([^18^F]RO5263397) as a fluorine-18 labeled PET tracer suitable for imaging peripheral TAAR1 expression. [^18^F]TAAR1-2203 was developed based on a previously identified 2-aminooxazoline-based TAAR1 agonist scaffold with high potency and selectivity for TAAR1 [37].

## Materials and methods

Solvents used for the radiosyntheses were purchased in anhydrous grade (puriss. dried over molecular sieves, H_2_O < 0.005%). Blocking agents RO5333943 (mouse TAAR1 Ki = 1.1 nM, rat TAAR1 Ki = 0.3 nM) and RO5425754 (mouse TAAR1 Ki = 0.6 nM, rat TAAR1 Ki = 0.2 nM) were kindly provided by F. Hoffmann-La Roche Ltd. The radioactivity of all samples was quantified using a Capintec CRC^®^−15R radioisotope dose calibrator. Purification of the radioligands was performed by semipreparative HPLC using a Merck Hitachi LaChrome L-7100 pump system, equipped with a Shimadzu SPD-10AV UV-vis detector and a Carroll & Ramsey Associates Model 105-S radiation detector, operated with PowerChrom software (version 2.7.9). A Phenomenex Luna C18 column (5 μm, 10 mm i.d. × 250 mm) was employed, using an isocratic eluent composed of water and CH₃CN (v/v = 20:80, with 0.1% triethylamine (TEA)) at a flow rate of 5 mL/min for 30 min, with UV detection at 254 nm. For quality control, an aliquot of the final formulated product was analyzed by analytical HPLC using a LabAlliance Series III pump system, a Carroll & Ramsey Associates Model 105-S radiation detector, and a Waters 2487 Dual λ Absorbance Detector UV-vis detector, operated with PowerChrom software (version 2.7.9). Analytical separations were performed using an XBridge BEH C18 column (3.5 μm, 4.6 mm × 150 mm) with an isocratic eluent of water and CH₃CN (v/v = 40:60, with 0.1% TEA), at a flow rate of 1 mL/min and UV detection at 254 nm (retention time = 4.6 min). Molar activity was determined by comparison to a standard curve generated with non-radioactive TAAR1-2203. Wild-type animals were purchased from Charles River, whereas TAAR1 knock-out animals and respective genetically matched controls were kindly provided by F. Hoffmann-La Roche Ltd. All animal studies were approved and conducted in accordance with the guidelines of the Institutional Animal Care and Use Committee (IACUC).

### Radiochemistry

[^18^F]Fluoride ions were generated via the nuclear reaction ^18^O(p, n)¹⁸F in a cyclotron (18 MeV protons, ^18^O-water, > 98%). An anion-exchange cartridge (Sep-Pak QMA Plus Light; Waters, Cat. No. 186004540) was used to trap and purify the [^18^F]fluoride ions, which were subsequently eluted with a solution of TBAOMs (3 mg) in MeOH (1 mL). The methanol was evaporated at 110 °C under a flow of helium (10 min). The radiosynthesis was performed in two steps, consisting of an initial nucleophilic radiofluorination followed by Boc-group deprotection under acidic conditions. For the radiofluorination step, the respective boronic ester precursor (2 mg) was dissolved in DMAc/n-BuOH (200/100 µL) together with TEAHCO_3_ (0.5 mg) and Cu(OTf)_2_(py)_4_ (7 mg), and the mixture was heated at 90 °C for 20 min. After cooling to ambient temperature, a solution of HCl in 1,4-dioxane (4 M, 200 µL) was added, and the reaction mixture was heated at 40 °C for 10 min. The reaction mixture was subsequently neutralized with NaHCO_3_ (0.8 mL, 1 M) and diluted with water. The resulting solution was loaded onto a Sep-Pak Plus C18 cartridge (Waters, Cat. No. WAT023515), and the product mixture was eluted with acetonitrile (1 mL). For purification, 2 mL of water was added, and the solution was injected into a semipreparative HPLC system. The collected product fraction was diluted with 10 mL of water and loaded onto an activated Sep-Pak Plus C18 cartridge (Waters, Cat. No. WAT023515), washed with 10 mL of water, and eluted with ethanol (0.5 mL). The final formulation consisted of the radiotracer in saline containing approximately 5% ethanol.

### Stability in formulation and distribution coefficient (LogD)

The stability of [^18^F]TAAR1-2203 in phosphate-buffered saline (PBS) was assessed after 60 min of incubation at 37 °C. Analytical HPLC was performed using an XBridge C18 column (3.5 μm, 4.6 mm × 150 mm) under isocratic conditions with a mobile phase of MeCN/H_2_O (35:65, v/v) containing 0.1% triethylamine (TEA) at a flow rate of 1.0 mL/min.

The distribution coefficient (LogD) was determined using the shake-flask method. Briefly, 500 µCi (18.5 MBq) of [^18^F]TAAR1-2203 was added to a presaturated mixture of PBS (1.3 mL, pH 7.4) and n-octanol (3 mL). The mixture was vortexed for 5 min, after which 500 µL aliquots (*n* = 3) were transferred into separate tubes and centrifuged at 3500–4000 rpm for 5 min. Following centrifugation, 50 µL of the n-octanol phase and 500 µL of the aqueous phase were carefully sampled and transferred into pre-weighed gamma counter tubes. After weighing, radioactivity in each phase was quantified using a Packard Cobra 5002/5003 gamma counter. The LogD value at pH 7.4 was calculated based on the measured weights and the radioactivity distribution between the organic and aqueous phases, as previously reported [38].

### Serum stability

The stability of [^18^F]TAAR1-2203 was assessed in mouse, rat, NHP, and human serum. Aliquots (400 µL) of serum were preincubated at 37 °C for 5 min, followed by the addition of [^18^F]TAAR1-2203 (20 µL, 300 µCi/11.1 MBq). After 60 min, 200 µL of each sample was collected, quenched with ice-cold MeCN (200 µL), centrifuged at 10,000 g for 5 min, and analyzed by radio-HPLC (XBridge C18 column, 3.5 μm, 4.6 mm × 150 mm; CH_3_CN/H_2_O 35:65 with 0.1% TEA; flow rate 1.0 mL/min). Experiments in PBS served as negative controls, and benfluorex hydrochloride was used as a positive control.

### Microsomal stability

Microsomal stability of [^18^F]TAAR1-2203 was evaluated using mouse, rat, NHP, and human liver microsomes. Potassium phosphate buffer (340 µL, 0.5 M, pH 7.4) was combined with NADPH regenerating solution (40 µL, 10 mM). Radiotracer solution (10 µL, 150 µCi, 5.5 MBq) was added, and the mixture was preincubated at 37 °C for 5 min. Liver microsomes (20 µL) were subsequently added. Incubations were carried out at 37 °C with gentle shaking. At 30 and 60 min, 200 µL aliquots were quenched with ice-cold MeCN (200 µL), centrifuged at 10,000 g for 5 min, and analyzed using an AR-2000 radio-TLC imaging scanner (Eckert & Ziegler).

### Plasma protein binding

Plasma protein binding of [^18^F]TAAR1-2203 was determined in mouse, rat, NHP, and human plasma. Plasma samples (150 µL, *n* = 3) were preincubated at 37 °C for 5 min before addition of [^18^F]TAAR1-2203 (10 µCi, 0.37 MBq). After 10 min of incubation, 300 µL of ice-cold PBS were added. Samples (300 µL) were then transferred onto Amicon^®^ centrifugal filter units (10 kDa cut-off) and centrifuged at 2100 g for 15 min at 4 °C. Filtrates were washed with 300 µL PBS and centrifuged again. Radioactivity in the filtrate and protein-bound fractions was measured using a Packard Cobra 5002/5003 gamma counter.

### In vitro autoradiography

In vitro autoradiography experiments with [^18^F]TAAR1-2203 were conducted on mouse and rat pancreas, kidney and brain tissue sections, respectively. Tissue Sect. (20 μm) were thawed on ice for 10 min and preincubated in assay buffer (50 mM Tris, pH 7.4) for 10 min. Subsequently, sections were incubated with [^18^F]TAAR1-2203 for 40 min at ambient temperature. For blocking studies, structurally distinct TAAR1 agonists, RO5333943 or RO5425754, were added in excess to the assay buffer at a final concentration of 10 µM. After incubation, sections were washed in assay buffer for 2 min on ice and dipped briefly in water (2 × 5 s), followed by drying and exposure to a phosphor imager plate for 60 min. Imager plates were scanned on an Amersham Typhoon analyzer system, and images were analyzed using ImageQuant TL 8.1, as previously reported [39].

### Ex vivo biodistribution

Female CD1 mice (16 weeks old, *n* = 4) received [^18^F]TAAR1-2203 (15 µCi, 0.56 MBq in 100 µL saline) via tail vein injection. For blocking studies, TAAR1 agonist RO5425754 (4 mg/kg) dissolved in saline containing 10% DMSO and 5% Tween^®^ 80 was administered shortly before tracer injection through the same i.v. catheter. Mice were sacrificed at 5, 15, 30, and 60 min post-injection (*n* = 4 per group), and organs were harvested, weighed, and measured for radioactivity using a Packard Cobra 5002/5003 gamma counter. Data were decay-corrected and expressed as percentage of injected dose per gram of wet tissue (%ID/g).

### Ex vivo radiometabolite analysis

Female CD1 mice (20 weeks old, *n* = 3) were injected with [^18^F]TAAR1-2203 (100 µCi, 3.7 MBq) and sacrificed 30 min post-injection. Brain, pancreas, and plasma samples were collected. Organs were homogenized in ice-cold acetonitrile (CH_3_CN). Plasma was obtained by centrifugation at 1400 rpm for 5 min at 4 °C, and proteins were precipitated with repeated CH_3_CN extraction until clear supernatants were obtained. Analytical samples were spiked with non-radioactive TAAR1-2203 and analyzed by radio-HPLC (XBridge C18 column; CH_3_CN/H₂O 35:65, with 0.1% TEA; flow rate 1.0 mL/min). Parent tracer fractions were determined as the percentage of intact [^18^F]TAAR1-2203 relative to total detected radioactivity.

### Receptor occupancy

Ex vivo receptor occupancy studies were conducted in female Sprague Dawley rats (20 weeks old). TAAR1 agonist RO5425754 was administered at doses of 0.05, 0.1, 0.3, 0.5, 1, 2, or 3 mg/kg (formulated in saline with 10% DMSO and 5% Tween^®^ 80) administered shortly before tracer injection through the same i.v. catheter. Rats were sacrificed at 15 min post-injection, and the pancreas was harvested for radioactivity measurement. Results were expressed as %ID/g and fitted using the following equation:

1 $$\frac{\%ID}g=\left(\frac{\%ID}g,max-\frac{\%ID}g,min\right)\ast\frac{D_{50}}{\left(D_{50}+d\right)}+\frac{\%ID}g,min\\$$

where ***%ID/g*** is the percentage of injected dose per gram wet tissues; ***%ID/g***,*** max*** is the upper plateau; ***%ID/g***,*** min*** is the lower plateau, ***D***_***50***_ represents the dose required for 50% receptor occupancy. Receptor occupancy (RO) in percent was calculated as ***d/(D***_***50***_ ***+ d)*** * 100 by rearranging Eq. 1.

### PET imaging

Dynamic PET imaging was performed for 60 min in female C57BL/6 mice, as well as in TAAR1 knock-out mice and respective controls, under 1–2% (v/v) isoflurane anaesthesia as previously reported [39–41]. [^18^F]TAAR1-2203 (1.5 MBq in 100 µL saline) was administered via tail vein injection. For blocking studies, TAAR1 agonist RO5425754 (4 mg/kg) was injected 5 min prior to radiotracer administration. Dynamic PET scans were acquired using a G8 PET/CT scanner (Sofie Biosciences, Culver, CA, USA) and analyzed with PMOD software (PMOD Technologies Ltd., Zurich, Switzerland). Radioactivity was decay-corrected and expressed as standardized uptake values (SUVs), normalized to body weight and injected dose.

### Statistical analyses

Data are presented as mean ± standard deviation (SD) unless otherwise stated. Comparisons between two groups were performed using the student’s t test, and comparisons across more than two groups were conducted using one-way ANOVA test followed by multiple comparisons Tukey’s post-hoc test when appropriate. Exact p-values are reported in the figure panels. A two-tailed p-value < 0.05 was considered statistically significant. Statistical analyses were conducted using GraphPad Prism version 9.0 (GraphPad Software, San Diego, CA).

## Results

Binding affinity studies with non-radioactive TAAR1-2203 (RO5263397) revealed high binding affinity for TAAR1 across species, with K_i_ values of 0.9 nM (mouse), 7.4 nM (rat), 22.5 nM (cynomolgus monkey), and 5.7 nM (human). Initial physicochemical profiling indicated that TAAR1-2203 possesses a favorable property profile for radiotracer development, with molecular weight, polar surface area (PSA), aqueous solubility, logD, LIMBA score, and PAMPA permeability values falling within the optimal range for both central and peripheral imaging applications (Supplemental Table 1). ADME studies confirmed that TAAR1-2203 exhibits a relatively high metabolic stability in rodent and human liver microsomes and hepatocytes (Supplemental Table 2). Of note, TAAR1-2203 was not a substrate of mouse or human P-glycoprotein (P-gp), as evidenced by low apical efflux ratios (1.0 in mouse, 1.2 in human), and demonstrated high apparent permeability (P_app_ >300 nm/s). In addition, plasma protein binding assays revealed a consistent free fraction of approximately 60% in rodent and human plasma. RO5263397 was well tolerated and did not exhibit any adverse events at doses up to 3 mg/kg and 10 mg/kg in dogs and rats, respectively, in 4-week repeat dose toxicity studies. Based on this favorable profile, a radiofluorinated analog was envisioned for the purpose of TAAR1-targeted PET imaging.

**Fig. 1:**
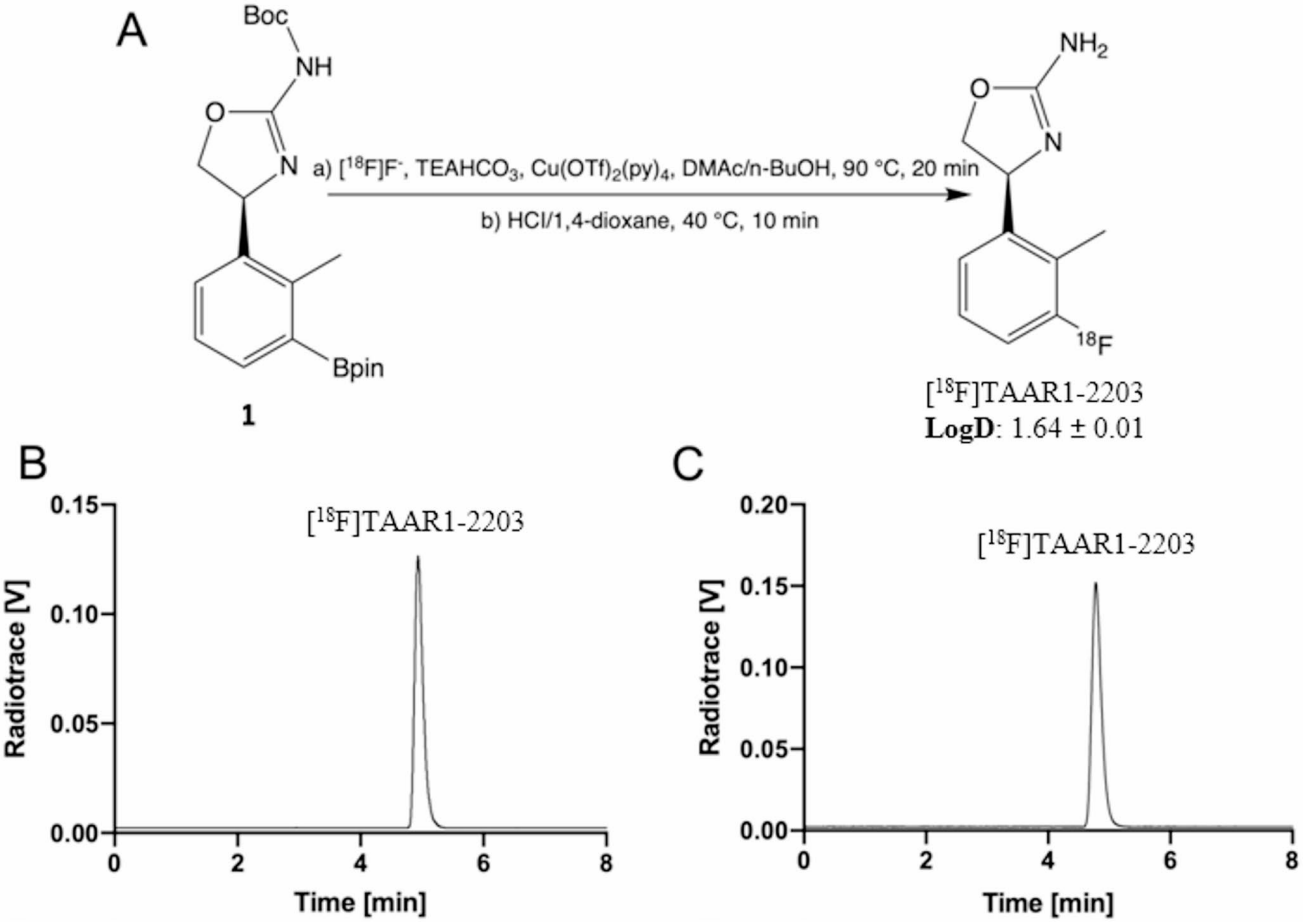
**(A)** Radiosynthesis of [^18^F]TAAR1-2203 from boronic ester precursor 1. A LogD_7.4_ value of 1.64 ± 0.01 (*n* = 3) was determined by the shake-flask method. **(B)** Stability of [^18^F]TAAR1-2203 after 60 min of incubation in phosphate buffer at 37 °C, showing no detectable degradation. **(C)** Stability of [^18^F]TAAR1-2203 after 60 min of incubation in saline (0.9% (w/v) sodium chloride in water) at 37 °C, indicating no detectable degradation

The boronic ester precursor 1 and the reference compound, TAAR1-2203, were synthesized as outlined in the Supporting Information. [^18^F]TAAR1-2203 was successfully obtained via a two-step procedure, as depicted in Fig. 1A. The synthesis involved an initial copper-mediated radiofluorination step, followed by acid-catalyzed Boc-group deprotection. [^18^F]TAAR1-2203 was obtained with a radiochemical conversion (RCC) of 49.5%, a radiochemical purity exceeding 99%, and a molar activity > 30 GBq/µmol. To assess suitability for extended PET imaging applications, the stability of [^18^F]TAAR1-2203 was evaluated in formulation for up to 4 h at ambient temperature, showing no detectable degradation. In addition, the non-radioactive reference compound TAAR1-2203 was incubated in plasma from mouse, rat, non-human primate, and human sources at 37 °C for up to 5 h. No degradation was observed in any species, further confirming the favorable chemical and metabolic stability across preclinical and translational settings. Along this line, > 99% of intact parent compound detected after 60 min of incubation in both saline (0.9% (w/v) sodium chloride in water) and phosphate buffer at 37 °C (Fig. 1B and C). The distribution coefficient (logD_7.4_) was determined by the shake-flask method and measured as 1.64 ± 0.01.

**Fig. 2:**
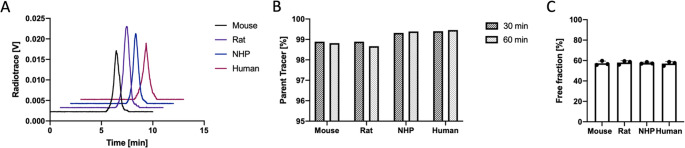
**(A)** HPLC analysis of [^18^F]TAAR1-2203 serum stability in mouse, rat, non-human primate (NHP), and human samples after 60 min of incubation at 37 °C, demonstrating only intact parent tracer with no detectable radiometabolites. **(B)** Stability of [^18^F]TAAR1-2203 in mouse, rat, NHP, and human liver microsomes at 30 and 60 min, expressed as percentage of intact parent tracer. **(C)** Plasma protein binding of [^18^F]TAAR1-2203 in mouse, rat, NHP, and human plasma, presented as percentage of free fraction. Plasma protein binding data are shown as mean percentage ± SD (*n* = 3)

The stability of [^18^F]TAAR1-2203 was further assessed in vitro using serum and liver microsomes. Serum stability studies were conducted in mouse, rat, NHP and human samples to evaluate the susceptibility of the tracer to metabolism and degradation. Following 60 min of incubation at 37 °C, > 99% of intact parent tracer was detected across all tested species, indicating good stability of [^18^F]TAAR1-2203 in plasma across species (Fig. 2A). Similarly, liver microsomal stability was evaluated in the same set of species to assess potential hepatic biotransformation. In rodent microsomes, > 98% of intact tracer was observed after 60 min, whereas in NHP and human microsomes, tracer integrity remained above 99% (Fig. 2B). These observations suggest that [^18^F]TAAR1-2203 exhibits a high metabolic stability in the presence of enzymatic systems implicated in oxidative metabolism.

The conserved stability profile across species supports the translational utility of [^18^F]TAAR1-2203 for preclinical and clinical investigations. Moreover, plasma protein binding studies revealed that [^18^F]TAAR1-2203 exhibited a free fraction of approximately 60%, which was consistent in all tested species (Fig. 2C). The relatively high free fraction implies that a substantial proportion of [^18^F]TAAR1-2203 remains unbound and available for receptor binding in vivo, which is advantageous for achieving adequate target signal during molecular imaging with PET. Collectively, these findings indicate that [^18^F]TAAR1-2203 possesses a suitable stability profile, supporting its potential for in vivo studies.

**Fig. 3:**
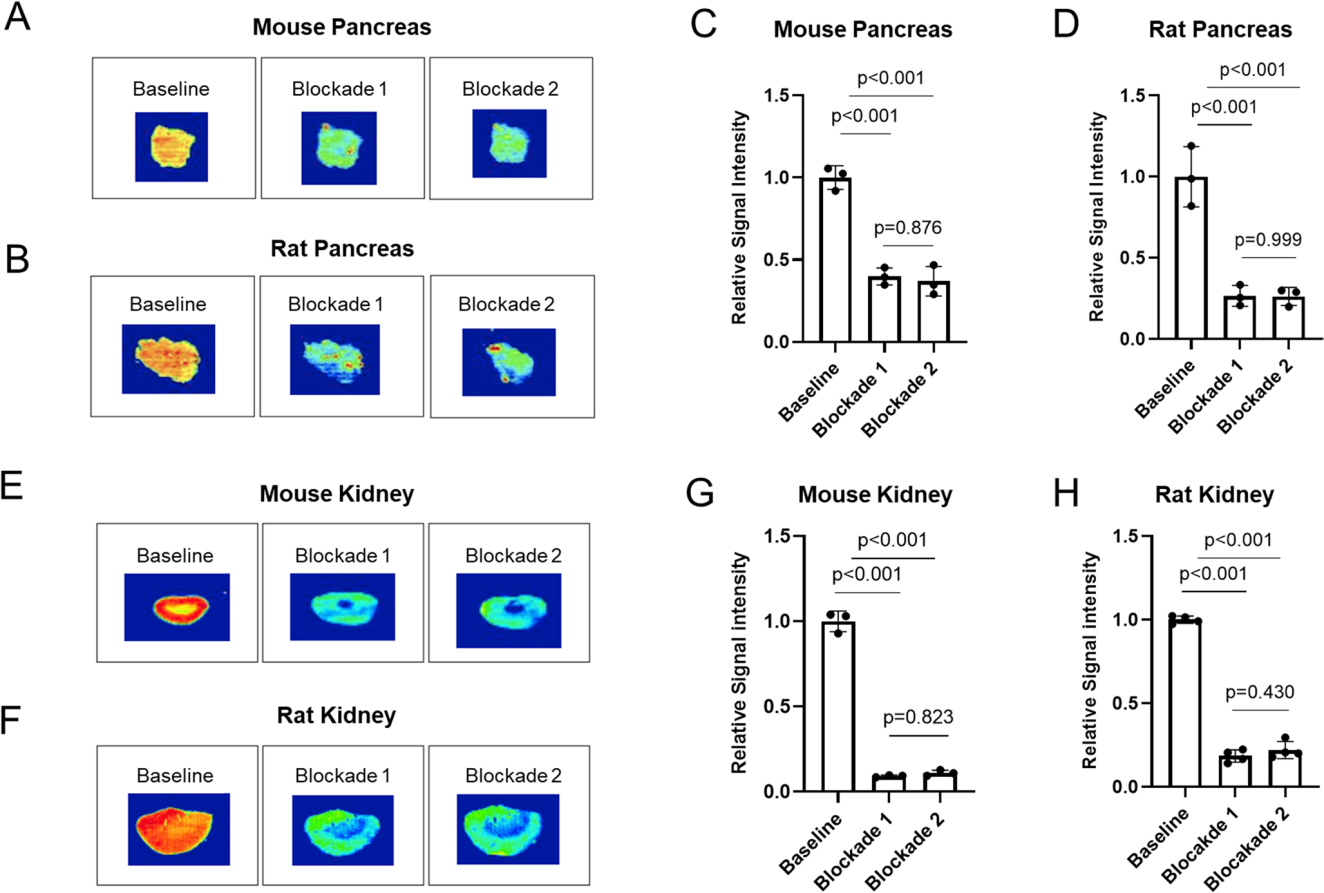
In vitro autoradiography of [^18^F]TAAR1-2203 with rodent pancreas and kidney tissue sections – peripheral organs that express endogenous TAAR1 and serve to assess the specificity of [^18^F]TAAR1-2203. Baseline conditions include [^18^F]TAAR1-2203 (5–8 nM) in the absence of any blockers. Blockades 1 and 2 contain [^18^F]TAAR1-2203 (5–8 nM) and one of the non-radioactive TAAR1 agonists RO5333943 or RO5425754, respectively, as blockers and in excess (10 µM). (**A**) Representative autoradiograms of the mouse pancreas. (**B**) Representative autoradiograms of the rat pancreas. (**C**) Quantification for the mouse pancreas. (**D**) Quantification for the rat pancreas. (**E**) Representative autoradiograms of the mouse kidney. (**F**) Representative autoradiograms of the rat kidney. (**G**) Quantification for the mouse kidney. (**H**) Quantification for the rat kidney. Data is presented as mean ± SD (*n* = 3)

To assess the binding specificity of [^18^F]TAAR1-2203, in vitro autoradiography was performed on pancreas and kidney sections derived from wild-type Sprague Dawley rats and CD1 mice (Fig. 3), respectively. The pancreas and kidney physiologically express TAAR1, thereby serving as potential tissues for the evaluation of tracer specificity [18].

Under baseline conditions, both species exhibited a robust binding signal of [^18^F]TAAR1-2203, which was markedly attenuated under blocking conditions. Specificity was confirmed by co-incubation with excess amounts of structurally distinct TAAR1 agonists, RO5333943 and RO5425754, at a final assay concentration of 10 µM. Quantitative analysis revealed a reduction of [^18^F]TAAR1-2203 binding by more than 50% in the pancreas and by greater than 70% in the kidney in both mouse and rat tissue sections. These findings indicate that [^18^F]TAAR1-2203 binds specifically to TAAR1 in peripheral tissues characterized by significant receptor expression and suggests the utility of pancreas and kidney as potential target organs for evaluating the target specificity of TAAR1-tailored probes.

**Fig. 4:**
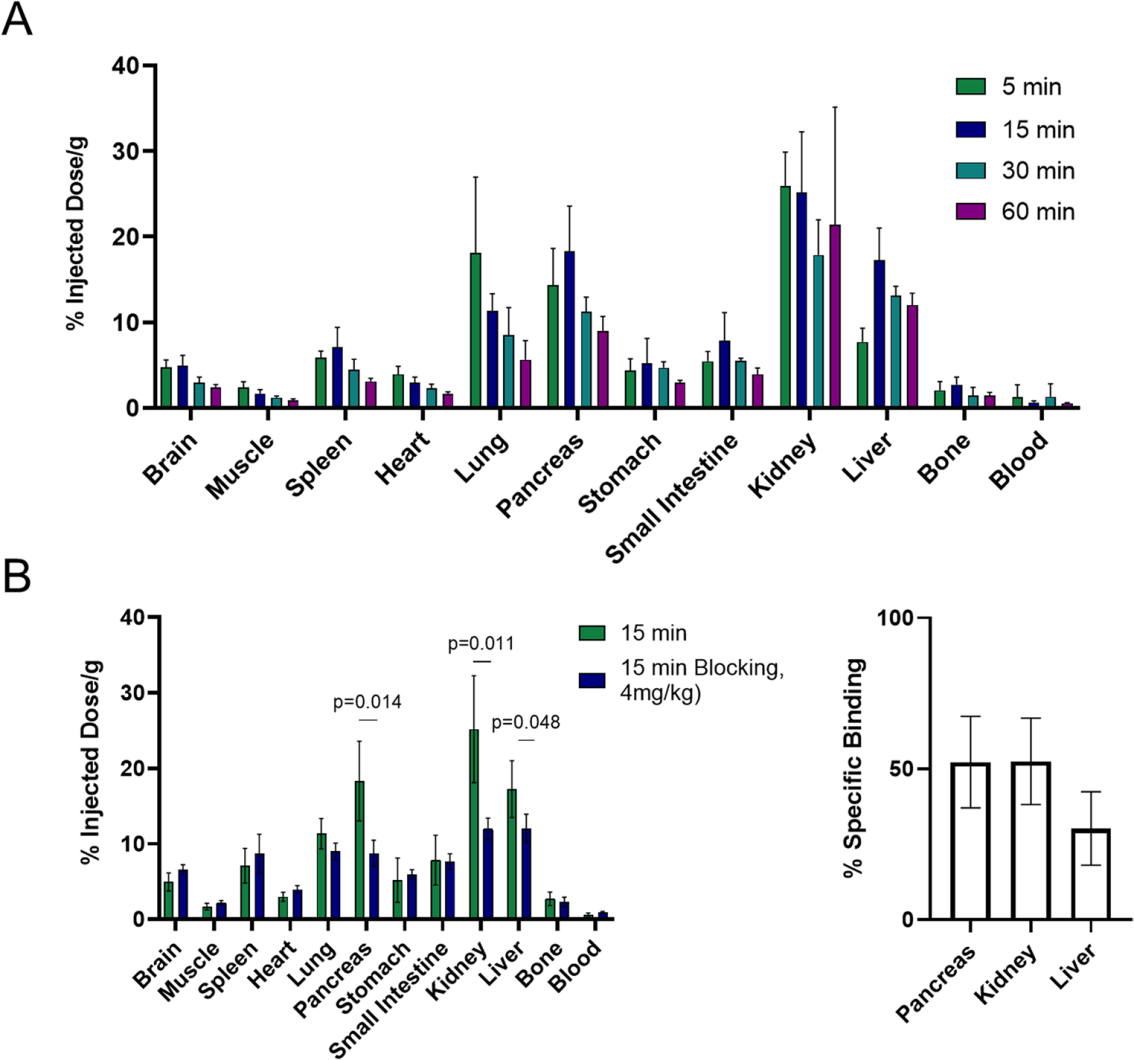
Ex vivo whole-body biodistribution of [^18^F]TAAR1-2203 in CD1 mice. (**A**) Ex vivo biodistribution under baseline conditions at 5, 15, 30 and 60 min post injection. (**B**) Ex vivo biodistribution of [^18^F]TAAR1-2203 in CD1 mice under baseline and blockade condition at 15 min post injection. The blocking group was injected with a non-radioactive TAAR1 agonist (RO5425754) at a dose of 4 mg/kg. Significant signal reduction was observed for the pancreas, kidney and liver. The results are expressed as percentage of the injected dose per gram of wet tissue (% ID/g) and presented as mean ± standard deviation (*n* = 4)

The pharmacokinetic properties of [^18^F]TAAR1-2203 were evaluated in a whole-body ex vivo biodistribution study conducted in female CD1 mice. To this end, the distribution of radioactivity across different tissues and organs was quantified at 5, 15, 30, and 60 min post-injection (*n* = 4 per time point) (Fig. 4A).

High initial uptake, defined as a percentage of injected dose per gram tissue (% ID/g), was observed in the lung, pancreas, and kidney, exceeding 10% ID/g in all three organs. Notably, while a fast washout was observed at 15 min post injection in the lung, radioactivity washout from the pancreas and the kidney was slower, indicating tissue retention in these organs. A blocking study was performed to further assess the specificity of [^18^F]TAAR1-2203 in vivo. As such, RO5425754, a structurally distinct TAAR1 agonist, was administered at a dose of 4 mg/kg prior to tracer injection, and tracer uptake was evaluated at 15 min post-injection (Fig. 4B). Significant signal reductions were observed in the pancreas, kidney, and liver, all of which are organs with significant physiological TAAR1 expression. Indeed, quantitative analysis revealed a specific binding of 52.2 ± 15.2% in the pancreas. Similarly, specificity values of 52.5 ± 14.3% and 30.2 ± 12.2% were observed in the kidney and the liver, respectively (Fig. 4B), indicating TAAR1-mediated binding in these peripheral organs. In contrast, no specific binding was detected in the brain, a finding that was subsequently confirmed by PET imaging studies and by comparison of brain autoradiograms of TAAR1 knock-out mice and respective controls.

**Fig. 5:**
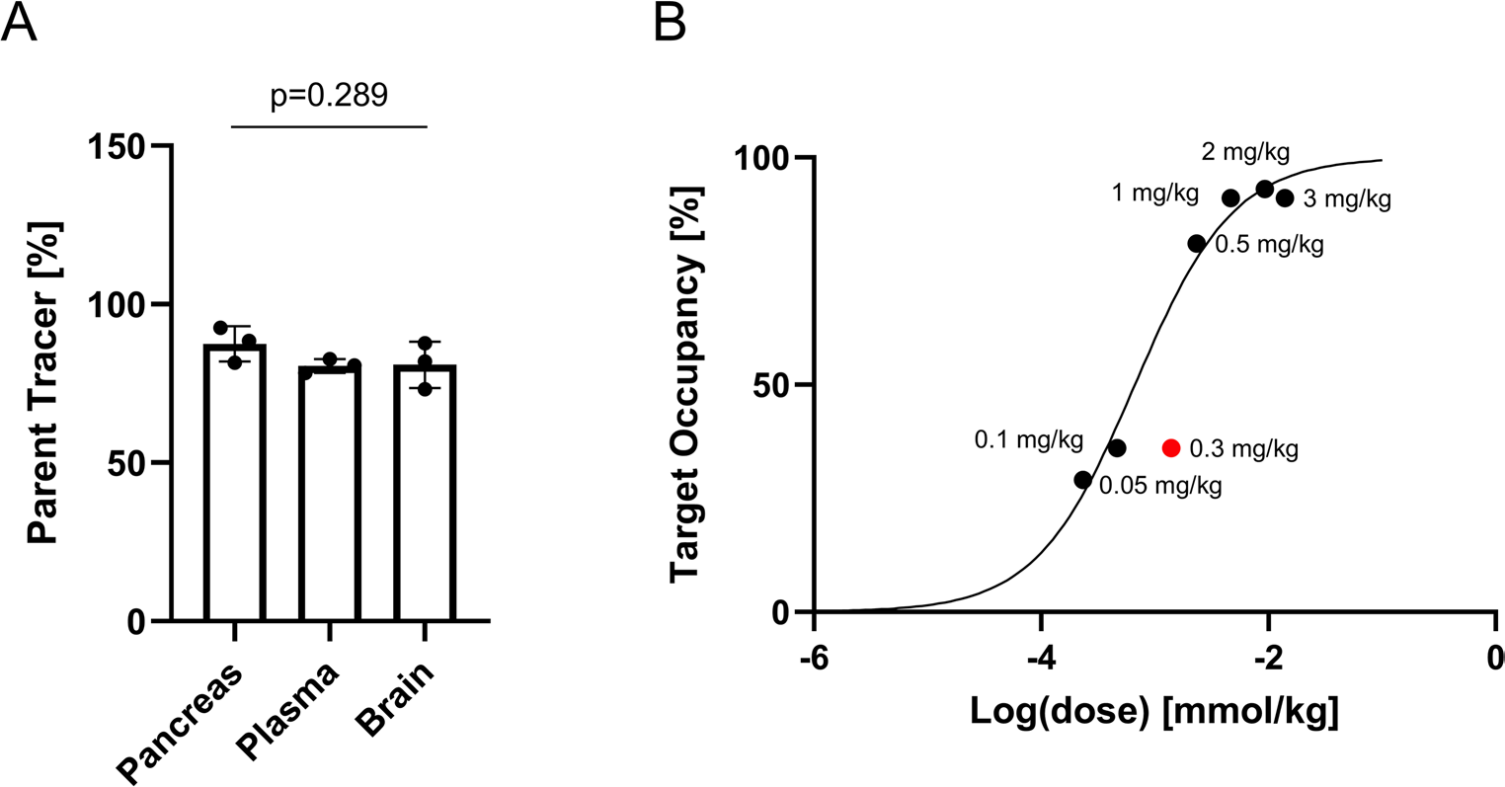
(**A**) Ex vivo radiometabolite analysis of [^18^F]TAAR1-2203 in the mouse pancreas, plasma and brain at 30 min post injection. Results are presented as mean ± SD (*n* = 3) (**B**) Ex vivo receptor occupancy of [^18^F]TAAR1-2203 in the rat pancreas at blocking concentrations of TAAR1 agonist RO5425754 ranging from 0.05–3 mg/kg. The dose 0.3 mg/kg (red dot) was excluded from the fit. The model predicted a D_50_ of 0.67 µmol/kg (0.14 mg/kg) for RO5425754

To further corroborate the stability of [^18^F]TAAR1-2203, an ex vivo radiometabolite analysis was performed in CD1 mice (*n* = 3) (Fig. 5A). At 30 min post-injection, the parent tracer fraction exceeded 80% in all analyzed matrices, including pancreas, plasma, and brain. These results are consistent with previous in vitro findings and underscore the metabolic stability of [^18^F]TAAR1-2203 in vivo. To demonstrate the suitability of [^18^F]TAAR1-2203 for quantitative assessment of drug-receptor interactions, an ex vivo receptor occupancy experiment was conducted in Sprague Dawley rats. Animals received escalating intravenous doses of RO5425754 ranging from 0.05 to 3 mg/kg, and tracer binding in the pancreas was quantified 15 min post-injection (Fig. 5B).

Nonlinear regression analysis yielded a robust fit to the occupancy data, from which a D_50_ value of 0.67 µmol/kg was calculated – demonstrating the utility of [^18^F]TAAR1-2203 to assess peripheral receptor occupancy in the pancreas.

**Fig. 6:**
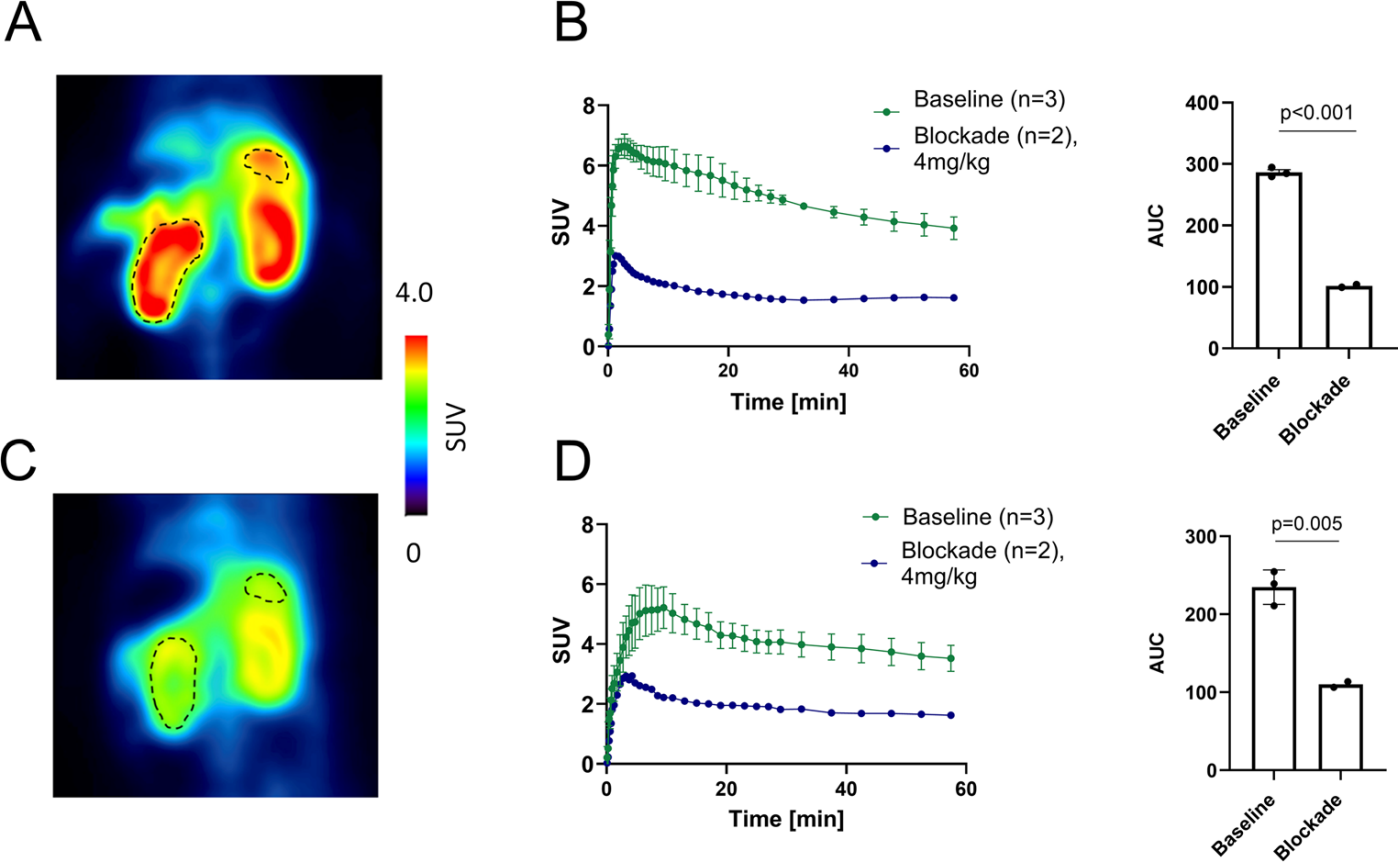
Positron Emission Tomography (PET) with [^18^F]TAAR1-2203 in CD1 mice. (**A**) Representative image for a baseline scan in the absence of a TAAR1 blocker, averaged from 0–60 min post injection. Kidney and pancreas are delineated. (**B**) Representative image of a blockade scan using 4 mg/kg (excess) of RO5425754 (averaged from 0–60 min). Kidney and pancreas are delineated. (**C**) Time activity curves of [^18^F]TAAR1-2203 in mouse kidney and quantification (0–60 min) of area under the curve for baseline vs. blockade scans. (**D**) Time activity curves of [^18^F]TAAR1-2203 in the mouse pancreas and quantification (0–60 min) of AUC for baseline vs. blockade scan. Results are presented as mean ± SD (*n* = 3) for baseline scans and mean (*n* = 2) for blockade scans

Dynamic PET imaging with [^18^F]TAAR1-2203 was performed in CD1 mice to evaluate tracer distribution and target specificity under baseline and blocking conditions. Representative coronal PET images, time-activity curves, and corresponding area under the curve (AUC) analyses for the kidney and pancreas are depicted in Fig. 6. High standardized uptake values (SUVs) were observed in both the kidney (Fig. 6C) and pancreas (Fig. 6D) under baseline conditions. Quantitative analysis of the AUC from 0 to 60 min post-injection revealed a significant reduction in tracer uptake following administration of TAAR1 agonist RO5425754 at the dose of 4 mg/kg, with significant blocking observed in the kidney and pancreas. These findings indicate that [^18^F]TAAR1-2203 exhibits high TAAR1 specificity in peripheral organs and may serve as a molecular imaging tool for non-invasive assessment of TAAR1 expression in kidney- and pancreas-related pathologies.

**Fig. 7:**
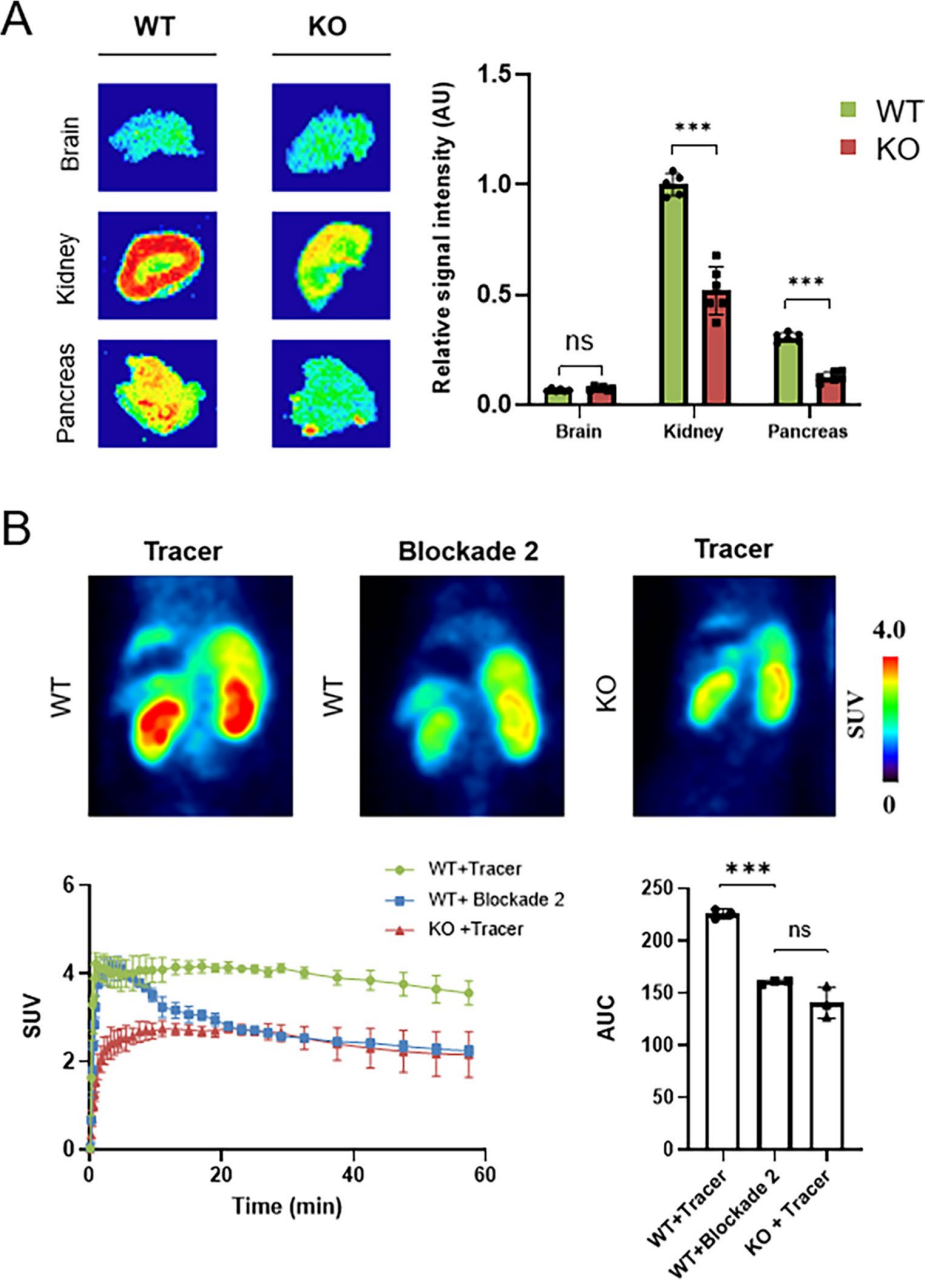
In vitro autoradiography and positron emission tomography (PET) of [^18^F]TAAR1-2203 in TAAR1 knockout (KO) mice and respective wildtype (WT) controls **(A)** Representative autoradiograms of [^18^F]TAAR1-2203 on TAAR1 KO mouse tissue sections, signal quantification is depicted for the brain, kidney and pancreas. Data is presented as mean ± SD (*n* = 6). (**B**) Representative PET image of a baseline [^18^F]TAAR1-2203 scan with WT and TAAR1 KO mice, averaged from 0–60 min post injection. The blocking scan was performed with [^18^F]TAAR1-2203 and 3 mg/kg (excess) of RO5425754 (0–60 min average). Time activity curves of [^18^F]TAAR1-2203 in mouse kidney and quantification of area under the curve (0–60 min). Results are presented as mean ± SD (*n* = 3)

To confirm whether the tracer binding observed in the kidney and pancreas is mediated by specific interactions with TAAR1, we conducted in vitro autoradiography and in vivo PET imaging studies in TAAR1 knockout (KO) mice and respective wild-type (WT) controls. As shown in Fig. 7A, in vitro autoradiography revealed a substantial reduction of [^18^F]TAAR1-2203 binding in the pancreas and kidney of TAAR1 KO animals, whereas tracer signal in brain tissue was comparably low in both WT and KO animals, respectively. Quantification of signal intensities demonstrated highly significant reductions in tracer binding in both the kidney (−50%, *p* < 0.001) and pancreas (−32%, *p* < 0.001) of KO animals, indicating that a large proportion of the [^18^F]TAAR1-2203 signal in these peripheral organs is attributable to specific TAAR1-mediated binding. To further substantiate these findings in vivo, dynamic PET scans were performed in WT and KO mice following intravenous injection of [^18^F]TAAR1-2203 (Fig. 7B). Tracer retention in the kidneys was markedly reduced in TAAR1 KO mice, closely resembling the degree of signal reduction observed under pharmacological blockade with an excess of RO5425754 (4 mg/kg). Time-activity curve analysis confirmed this observation and revealed a significant reduction in the area under the curve (AUC_0−60 min_) in KO animals compared to WT controls. Together, our data provide strong evidence that [^18^F]TAAR1-2203 binds to TAAR1 in vivo and underscores the utility of the tracer for assessing TAAR1 expression.

## Discussion

The present study aimed to develop the first suitable PET radioligand for imaging TAAR1 in vivo. While TAAR1 has garnered substantial interest as a therapeutic target in neuropsychiatric and metabolic disorders, non-invasive imaging tools enabling quantification of receptor expression and drug-receptor interactions have been lacking. Here, we report the development of [^18^F]TAAR1-2203, the first TAAR1-targeted PET radioligand with suitable in vitro and in vivo performance characteristics. We demonstrate that [^18^F]TAAR1-2203 enables the quantification of TAAR1 receptor occupancy and the non-invasive mapping of receptor abundance in peripheral organs, including the pancreas and kidney. While TAAR1 expression in the rodent brain was insufficient to support specific imaging, the novel probe offers a unique opportunity to interrogate TAAR1 function in metabolic disorders involving the pancreas, as well as in kidney-related pathologies.

TAAR1 is currently being evaluated as a therapeutic target in both CNS and metabolic disorders, and several TAAR1 agonists have advanced into clinical development. Along this line, [^18^F]TAAR1-2203 may serve as a valuable tool to support patient stratification and therapeutic monitoring. Non-invasive quantification of TAAR1 expression in peripheral organs such as the pancreas could enable identification of individuals with altered receptor function, which has been linked to metabolic disease and an increased susceptibility to type 2 diabetes [20, 42]. If validated in higher species, such a biomarker tool could be employed to guide patient inclusion criteria in early-phase trials of TAAR1-directed therapies. Further, PET imaging with [^18^F]TAAR1-2203 allows for in vivo assessment of receptor occupancy, which is a critical component of exposure-response profiling and can be leveraged to inform rational dose selection in drug development. Similarly, longitudinal imaging may enable the detection of potential treatment-induced alterations in TAAR1 expression. The ability to visualize and quantify such dynamics in vivo could provide important insight into treatment durability and receptor regulation, and ultimately help optimize chronic dosing regimens for TAAR1-targeted therapies. Notably, TAAR1 agonists have demonstrated antipsychotic- and anxiolytic-like properties and are currently under clinical evaluation for the treatment of schizophrenia and substance use disorders​ [8]. Conversely, TAAR1 antagonists have been proposed for the management of PD, where TAAR1 inhibition has been associated with increased dopaminergic neuron vulnerability and a diminished response to L-DOPA [15]. Apart from its role in neurodegenerative diseases, TAAR1 is expressed across a range of peripheral tissues, where it plays important roles in metabolic and immune regulation. In humans and rodents, robust TAAR1 expression has been detected in pancreatic β-cells, where its activation modulates insulin secretion and glucose homeostasis [36]. TAAR1 is also found throughout the gastrointestinal tract, including the stomach and duodenum, where it co-localizes with gut hormones such as GLP-1 and peptide YY, mediating incretin-like effects and delayed gastric emptying [43]. These functions are physiologically relevant for regulating food intake, body weight, and nutrient absorption. In the immune system, TAAR1 is expressed in multiple leukocyte subtypes including T cells, B cells, and granulocytes [44–46]. Activation of TAAR1 in these cells modulates chemotaxis, cytokine secretion (e.g., IL-4), and immunoglobulin production. In the kidney, TAAR1 has been shown to mediate vasoconstrictor responses through calcium influx, calcium sensitization and reactive oxygen species signaling [47]. These effects were abrogated by the selective TAAR1 antagonist, EPPTB, and are consistent with functional receptor expression in the kidney. Among these peripheral functions of TAAR1, the implications in metabolic disease is of particular interest. Although the molecular mechanisms remain unclear, TAAR1 was found to modulate insulin secretion from β-cells, as well as to play a role in gut motility, satiety and eating behaviors [19, 21]. Indeed, modulating TAAR1 signaling by agonists constitutes an intriguing concept for anti-diabetic treatment [20].

Despite the advances in TAAR1-targeted drug development, there is only one report on a TAAR1-targeted PET ligand to date. Liang and co-workers recently labeled a structural analog of the TAAR1 antagonist, EPPTB, with carbon-11 and evaluated the probe in mice [34]. Notably, the previously reported TAAR1 PET ligand, [^11^C]TAAR1-1911, was developed based on a high-affinity TAAR1 antagonist and showed favorable in vitro properties including high binding affinity (Ki = 2.0 nM), physicochemical stability, and predicted BBB penetration [34]. However, [^11^C]TAAR1-1911 failed to show in vivo specificity due to rapid metabolic degradation and the accumulation of radiolabeled metabolites in the brain and plasma. Radiometabolite analysis indicated that only ~ 5% of intact parent compound was present in the brain at 30 min post-injection, limiting its utility for quantitative PET imaging. Furthermore, blocking studies with both the non-radioactive reference compound and the selective TAAR1 antagonist, EPPTB, failed to demonstrate displaceable binding. In the present study, we aimed to address this gap by developing a more stable PET radioligand with suitable pharmacokinetics for in vivo imaging. [^18^F]TAAR1-2203 showed high metabolic stability in vivo, enabled specific binding in peripheral tissues, and allowed in vivo receptor occupancy assessment by PET. These improvements address the key limitations of [^11^C]TAAR1-1911 and underscore the translational potential [^18^F]TAAR1-2203 as a promising tool to facilitate TAAR1-targeted drug development.

[^18^F]TAAR1-2203 was synthesized with high radiochemical purity, molar activity and exhibited excellent metabolic stability in microsomal incubations across different species. Notably, > 80% of the parent compound was detectable in plasma, pancreas, and brain at 30 min post-injection in vivo, thus offering a decent time window for fluorine-18 based PET imaging. The absence of increasing bone signal over time further confirmed the absence of significant in vivo defluorination. TAAR1-specific binding of [^18^F]TAAR1-2203 was confirmed in distinct peripheral tissues with notable physiological TAAR1 expression, including the kidney and pancreas [18, 48, 49], by in vitro autoradiography, ex vivo biodistribution, and PET imaging. The tracer displayed high signal intensity in these organs under baseline conditions, which was markedly reduced under pharmacological blockade or in TAAR1 knockout animals. These findings provide converging evidence for specific tracer–receptor interactions and position [^18^F]TAAR1-2203 as a suitable tool for assessing peripheral TAAR1 distribution and drug occupancy in vivo. In contrast, tracer accumulation in the brain remained unaffected by either blocking or TAAR1 deletion, suggesting that TAAR1 expression in the healthy rodent brain may fall below the detection threshold for PET imaging with [^18^F]TAAR1-2203 – notwithstanding its subnanomolar affinity for mouse TAAR1. While [^18^F]TAAR1-2203 seems to be brain-penetrant, as evidenced by a relatively slow washout kinetics on brain time-activity curves and high intact parent fraction on ex vivo radiometabolite analysis, no specific TAAR1 binding was observed in the CNS by in vitro autoradiography or PET imaging, respectively. TAAR1-2203 exhibits high membrane permeability and is not a P-glycoprotein substrate, suggesting that the lack of specific TAAR1 signal in the brain is not due to poor pharmacokinetics. Of note, there was no distinction between the autoradiograms of TAAR1 knock-out brain tissue sections and respective wild-type controls, indicating that the probe is not sensitive to the basal TAAR1 expression levels in the mouse brain. This interpretation is supported by previous reports describing moderate baseline TAAR1 expression in wild-type rodent brains [12, 35]. Our findings highlight the challenges of imaging CNS TAAR1 under baseline conditions, while peripheral data indicates that [^18^F]TAAR1-2203 may potentially be employed as a surrogate marker for CNS occupancy via peripheral tissues – most notably, the pancreas, where TAAR1-specific signal was robust and quantifiable in our study. Further, TAAR1-2203 provides a promising scaffold for the development of next-generation ligands with further enhanced potency, with the goal of achieving a favorable B_max_/K_D_ ratio sufficient for CNS-targeted imaging. While the current study focused on establishing tracer specificity, metabolic stability, and peripheral binding profile, we acknowledge that full quantitative modeling was not performed. Future studies will implement kinetic modeling using a metabolite-corrected arterial input function to derive quantitative parameters such as volume of distribution and binding potential, as well as to support more advanced models for receptor occupancy assessment. Such modeling will be critical for establishing robust exposure-occupancy relationships for TAAR1 agonists currently in development. The application of compartmental modeling further holds potential to aid in validating peripheral tissues, such as the pancreas, as surrogate sites for CNS target engagement. The ability to quantify drug–receptor interactions non-invasively paves the way for pharmacokinetic/pharmacodynamic (PK/PD) modeling of TAAR1 agonists in the pipeline, thereby support dose selection for early clinical trials. Further, considering that TAAR1 activation has been shown to increase glucose-dependent insulin secretion by pancreatic β-cells [18, 19, 48], thereby normalizing glucose excursion during an oral glucose tolerance test in mice [19], it is envisioned that [^18^F]TAAR1-2203 will facilitate research in the field by means of allowing non-invasive mapping of TAAR1 receptor abundance. [^18^F]TAAR1-2203 holds promise for clinical translation as a peripheral TAAR1 PET radioligand.

## Conclusion

Collectively, this work led to the discovery and preclinical validation of [^18^F]TAAR1-2203, the first TAAR1-targeted PET radioligand with favorable in vivo performance characteristics that allow TAAR1 mapping and target occupancy assessment. The tracer demonstrated high affinity, metabolic stability, and specific binding to TAAR1 in peripheral tissues, particularly in the pancreas and kidney. By enabling non-invasive receptor mapping and target occupancy studies, [^18^F]TAAR1-2203 constitutes a valuable molecular imaging tool for advancing TAAR1-targeted drug discovery. Future studies will focus on evaluating its performance characteristics in higher species and exploring its translational potential for clinical applications.

## Supplementary Information

Below is the link to the electronic supplementary material.

Supplementary Material 1 (DOCX 820 KB)

## Data Availability

The datasets generated or analyzed during this study are available from the corresponding author on reasonable request.
