# Supplementary material for: Preclinical development of [18F]TAAR1-2203 as a PET radioligand for imaging TAAR1 expression and receptor occupancy
Source: Eur J Nucl Med Mol Imaging. 2025 Nov 6;53(4):2540–53. doi: 10.1007/s00259-025-07564-w (PMC12920416; doi:10.1007/s00259-025-07564-w)
Supplement: Supplementary file 1 — Supplementary Material 1 (DOCX 820 KB) [file 259_2025_7564_MOESM1_ESM.docx]

**Preclinical Development of [^18^F]TAAR1-2203 as a PET Radioligand for Imaging TAAR1 Expression and Receptor Occupancy**

Achi Haider,^1,2†^ Zhiwei Xiao,^1,3,4†^ Jiahui Chen,^1,3†^ Stefanie K. Pfister,^1,5^ Xin Zhou,^3^ Yinlong Li,^1,3^ Ahmad Chaudhary,^3^ Chunyu Zhao,^1,3^ Jian Rong,^1,3^ Axel Paehler,^2^ Susanne Mohr,^2^ Roger D. Norcross,^2^ Michael Honer,^2^ Linjing Mu,^5^ Luca Gobbi,^2^ Roger Schibli,^5^ Marius C. Hoener,^2^ Steven H. Liang^1,3,^*

^1^ Department of Radiology, Division of Nuclear Medicine and Molecular Imaging Massachusetts General Hospital and Harvard Medical School, 55 Fruit Street, Boston, MA 02114, USA.

^2^ Pharma Research and Early Development, Roche Innovation Center Basel, F. Hoffmann-La Roche, 4070 Basel, Switzerland.

^3^ Department of Radiology and Imaging Sciences, Emory University, 1364 Clifton Road, Atlanta, GA 30322, USA.

^4^ Department of Nuclear Medicine, Zhongnan Hospital of Wuhan University, Wuhan, China.

^5^ Center for Radiopharmaceutical Sciences ETH-PSI-USZ, Institute of Pharmaceutical Sciences ETH, Vladimir-Prelog-Weg 4, 8093 Zurich, Switzerland.

^†^Equal contribution

*Corresponding author: Steven H. Liang: [steven.liang@emory.edu](mailto:steven.liang@emory.edu)

**Supporting Information**

**Chemistry**

*(S)-2-(3-bromo-2-methylphenyl)-2-(((S)-1-(4-methoxyphenyl)ethyl)amino)acetonitrile hydrochloride (3)*

To a solution of (S)-1-(4-methoxyphenyl)ethanamine hydrochloride (2, 38.1 g, 203 mmol) and NaCN (10.3 g, 211 mmol) in H2O (40 mL) and MeOH (40 mL) was added 3-bromo-2-methyl-benzaldehyde (40 g, 201 mmol). The mixture was stirred at 25°C for 5 h and detected by TLC (Petroleum ether/EA=10/1, Rf=0.25). The mixture was then diluted with H2O (22.5 mL) and stirred at 25°C for 30 min. After filtrated and washed with H2O (9.0 mL), the residue was triturated with MeOH and filtered to afford a white solid. Compound 3 was obtained as a white solid (61.6 g, 77%). 1H NMR (400 MHz, DMSO-d6) δ 7.62 (d, J = 7.9 Hz, 1H), 7.57 (d, J = 7.6 Hz, 1H), 7.32 (d, J = 8.6 Hz, 2H), 7.22 (t, J = 7.9 Hz, 1H), 6.94 (d, J = 8.6 Hz, 2H), 4.44 (d, J = 11.4 Hz, 1H), 3.94 (qd, J = 6.4, 2.6 Hz, 1H), 3.75 (s, 3H), 2.13 (s, 3H), 1.30 (d, J = 6.5 Hz, 3H).

*(S)-2-amino-2-(3-bromo-2-methylphenyl)acetic acid hydrochloride (4)*

(S)-2-(3-bromo-2-methylphenyl)-2-(((S)-1-(4-methoxyphenyl)ethyl)amino)acetonitrile hydrochloride (3, 78.7 g, 199 mmol) was added HCl (6 M, 1.97 L). After stirring at 90°C for 4 h, the mixture was cooled to 25°C and extracted with MTBE (300 mL). The aqueous layer was collected and concentrated under reduced pressure to give the crude product. The crude product was used directly for the next step without further purification. Compound 4 was obtained as a white solid (63.0 g, crude). 1H NMR (400 MHz, CD3OD) δ 7.66-7.71 (m, 1H), 7.42 (d, J = 7.3 Hz, 1H), 7.20-7.26 (m, 1H), 5.45 (s, 1H), 2.61 (s, 3H).

*(S)-methyl 2-amino-2-(3-bromo-2-methylphenyl)acetate (5)*

(S)-2-amino-2-(3-bromo-2-methylphenyl)acetic acid hydrochloride (4, 63.0 g, 225 mmol) was added HCl/ MeOH (6 M, 315 mL). After stirring at 25°C for 6 h (detected with LCMS), the reaction mixture was diluted with 5 M NaHCO3 (200 mL) and extracted with EA (20 mL×3). The combined organic layers were dried with Na2SO4, filtered, and concentrated. The concentrated crude product was used directly for the next step without further purification. Compound 5 was obtained as a colorless oil (47.2 g, crude). 1H NMR (400 MHz, CD3OD) δ 7.52 (d, J = 8.0 Hz, 1H), 7.28 (d, J = 7.8 Hz, 1H), 7.06-7.12 (m, 1H), 4.90 (s, 1H), 3.69 (s, 3H), 2.51 (s, 3H). LCMS (m/z) calcd for 258.0, found 258.1 (M+H)+, tR = 0.318 min.

*(S)-2-amino-2-(3-bromo-2-methylphenyl)ethanol (6)*

To a solution of methyl (S)-methyl 2-amino-2-(3-bromo-2-methylphenyl)acetate (5, 47.0 g, 182 mmol) in THF (100 mL) was added LiAlH4 (6.91 g, 182 mmol). The mixture was stirred at 0°C for 0.5 h, then diluted with H2O (20 mL) and extracted with EA (50 mL x 3). The combined organic layers were dried over Na2SO4, filtered, and concentrated. The crude product was used directly for the next step without purification. Compound 6 was obtained as a colourless oil (40.0 g, crude). 1H NMR (400 MHz, CD3OD) δ 7.47 (d, J = 7.9 Hz, 1H), 7.42 (d, J = 7.9 Hz, 1H), 7.10 (t, J = 7.9 Hz, 1H), 4.23-4.34 (m, 1H), 3.66 (dd, J = 11.0, 4.5 Hz, 1H), 3.48 (dd, J = 10.9, 7.8 Hz, 1H), 2.47 (s, 3H).

*(S)-4-(3-bromo-2-methylphenyl)-4,5-dihydrooxazol-2-amine (7)*

To a solution of (S)-2-amino-2-(3-bromo-2-methylphenyl)ethanol (6, 40.0 g, 174 mmol) in MeOH (350 mL) was added BrCN (21.1 g, 199 mmol) and AcONa (14.3 g, 174 mmol) at 0°C. After stirring at 25 °C for 2 h (detected with LCMS), the mixture was diluted with H2O (50 mL) and extracted with EA (100 mL×3). The combined organic layers were dried over Na2SO4, filtered, and concentrated. The crude product was used directly for the next step without purification. Compound 7 was obtained as a colourless oil (40.0 g, crude). 1H NMR (400 MHz, CD3OD) δ 7.56 (d, J = 7.9 Hz, 1H), 7.38 (d, J = 8.0 Hz, 1H), 7.16 (d, J = 7.8 Hz, 1H), 4.65 (dd, J = 7.9, 4.8 Hz, 1H), 3.72-3.77 (m, 1H), 3.64-3.70 (m, 1H), 2.48 (s, 3H). LCMS (m/z) calcd for 255.0, found 255.0 (M+H)+, tR = 0.460 min.

*(S)-tert-butyl (4-(3-bromo-2-methylphenyl)-4,5-dihydrooxazol-2-yl)carbamate (8)*

To a solution of (S)-4-(3-bromo-2-methylphenyl)-4,5-dihydrooxazol-2-amine (7, 40.0 g, 157 mmol) in THF (400 mL) was added DMAP (23.0 g, 188 mmol) and (Boc)2O (17.1 g, 78.4 mmol) at 0 °C. The mixture was stirred at 0°C for 2 h and detected with LCMS. Then, the reaction mixture was diluted with H2O (50 mL) and extracted with EA (100 mL×3). The combined organic layers were dried over Na2SO4, filtered, and concentrated. The residue was purified by prep-HPLC (column: YMC Triart C18 250×50mm×7μm; CH3CN/ NH4HCO3aq=38-68%). Compound 8was obtained as a white solid (7.7 g, 14%). 1H NMR (400 MHz, CD3OD) δ 7.59 (d, J = 7.9 Hz, 1H), 7.15-7.20 (m, 1H), 7.07-7.12 (m, 1H), 5.68 (dd, J = 8.5, 4.1 Hz, 1H), 4.71 (t, J = 8.6 Hz, 1H), 4.06 (dd, J = 8.6, 4.1 Hz, 1H), 2.45 (s, 3H), 1.27 (s, 9H). LCMS (m/z) calcd for 355.1, found m/z = 354.9 (M+H)+, tR = 0.622 min.

*tert-butyl (S)-(4-(2-methyl-3-(4,4,5,5-tetramethyl-1,3,2-dioxaborolan-2-yl)phenyl)-4,5-dihydrooxazol-2-yl)carbamate (9)*

A mixture of (S)-tert-butyl (4-(3-bromo-2-methylphenyl)-4,5-dihydrooxazol-2-yl)carbamate (8, 2.1 g, 6 mmol), Boc2O (3.0 g, 12 mmol), Pd(dppf)Cl2 (865 mg, 1.2 mmol), AcOK (1.7 g, 18 mmol) in 1,4-dioxane (40 mL) was degassed and purged with N2 for 3 times, and then the mixture was stirred at 100°C for 3 h under N2 atmosphere. After detected with TLC (Petroleum ether/EA= /1, Rf=0.3), the reaction mixture was concentrated and purified by column chromatography (Petroleum ether/EA=10/1 to 3/7). Compound 9 was obtained as a brown solid (408 mg, 16.2%). 1H NMR (400 MHz, CDCl3) δ 7.70 (dd, J = 5.6, 1.8 Hz, 1H), 7.18-7.26 (m, 2H), 5.51 (dd, J = 8.0, 3.6 Hz, 1H), 4.57 (t, J = 8.4 Hz, 1H), 3.95 (dd, J = 8.0, 3.6 Hz, 1H), 2.49 (s, 3H), 1.34 (s, 12H), 1.24 (s, 9H). 13C NMR (101 MHz, CDCl3) δ 153.7, 149.3, 138.4, 136.2, 133.8, 124.3, 124.0, 81.9, 75.3, 67.6, 54.2, 25.8, 23.0, 22.9, 15.9. LCMS (m/z) calcd for 403.2, found m/z = 403.1 (M+H)+, tR = 2.037 min.

**Radiochemistry and Quality Control**


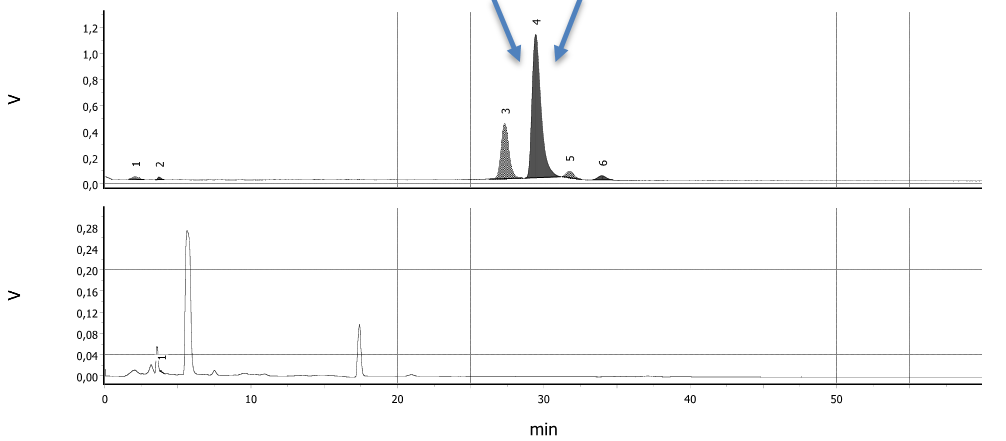


**Supplemental Figure 1**: Semipreparative HPLC for [^18^F]TAAR1-2203. Collected fraction indicated with blue arrows. The upper panel represents the radio-trace, whereas the lower panel is the UV-trace.


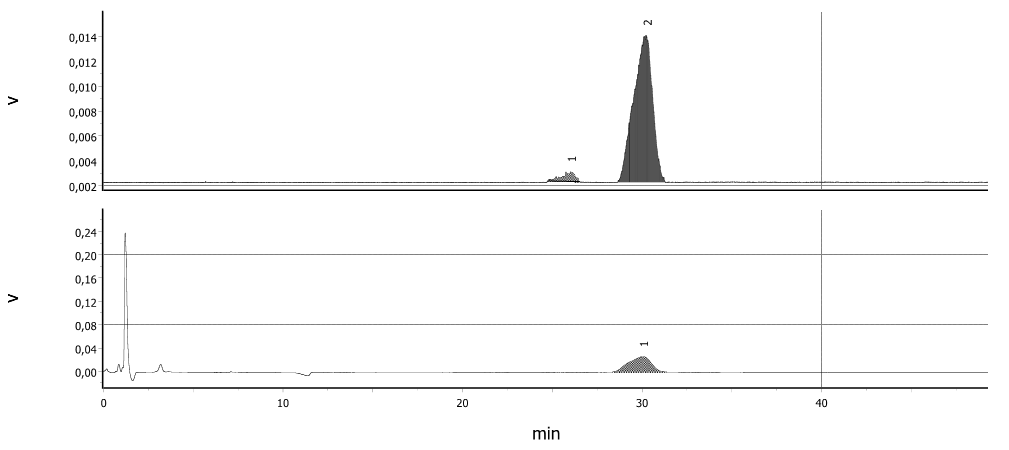


**Supplemental Figure 2**: Analytical HPLC for [^18^F]TAAR1-2203 including co-injection of non-radioactive TAAR1-2203. The upper panel represents the radio-trace, whereas the lower panel is the UV-trace.


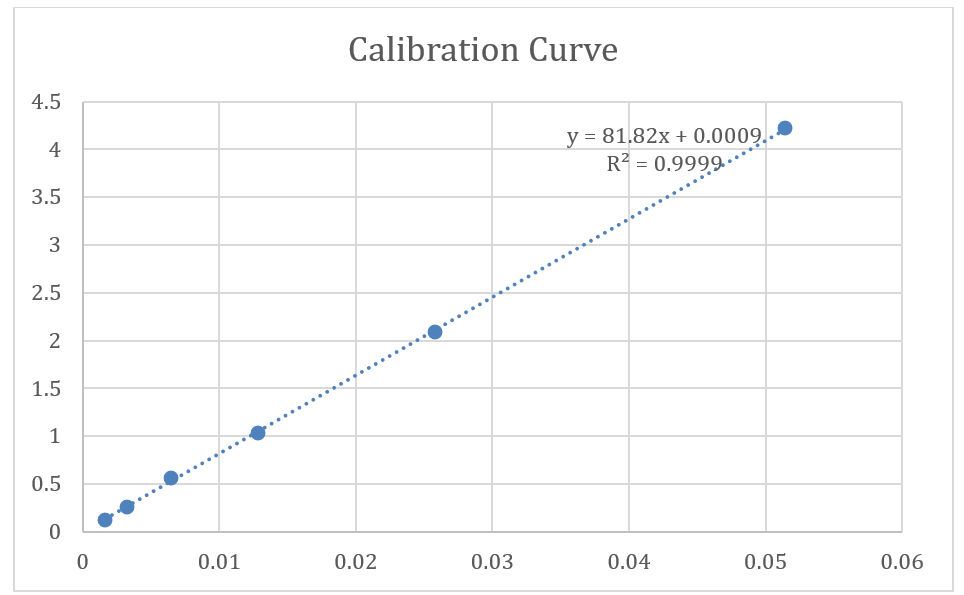


**Supplemental Figure 3**: Calibration curve for TAAR1-2203 based on Analytical HPLC. Equation was used to assess the molar activity following radiosynthesis of [^18^F]TAAR1-2203.

**Physiochemical and ADME Profiling of TAAR1-2203**

**Supplemental Table 1.** Physicochemical properties of TAAR1-2203.

| MW [g/mol] | 194.2 |
| --- | --- |
| PSA [Å^2^] | 42.5 |
| Solubility^a^ [µg/mL] | 210 |
| logD^b^ | 1.12 |
| PAMPA P_eff_ [cm/s*10E^-6^] | 14.5 |
| Chemical stability in aqueous buffer | Stable at pH 1, 4, 6.5 and 8 for 2 h at 37 °C |

^a^ Aqueous solubility at pH 6.5 in 0.05 M phosphate buffer; ^b^ pH 7.4

**Supplemental Table 2.** ADME profile of TAAR1 ligand TAAR1-2203.

| human / rat clearance in microsomes [µL/min/mg protein] | 14 / 10 |  |  |  |
| --- | --- | --- | --- | --- |
| human / rat clearance in hepatocytes [µL/min/10E6 cells] | 10 / 1.7 |  |  |  |
| human / rat plasma protein binding [%] | 62 / 61 |  |  |  |
| human / mouse P-glycoprotein efflux ratio  P_app, AB inhibitor_ [nm/s] | 1.2 / 1.0  412 / 316 |  |  |  |

**LogD Determination**

**Supplemental Table 3:** LogD determination for TAAR1-2203


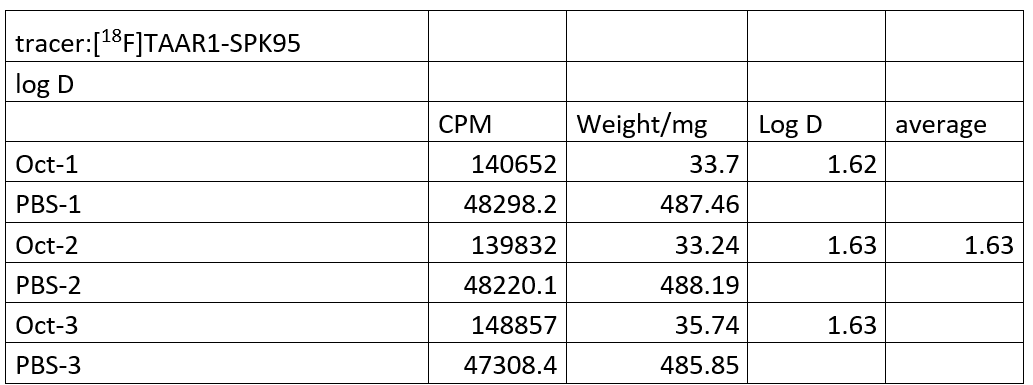


**PET Imaging**


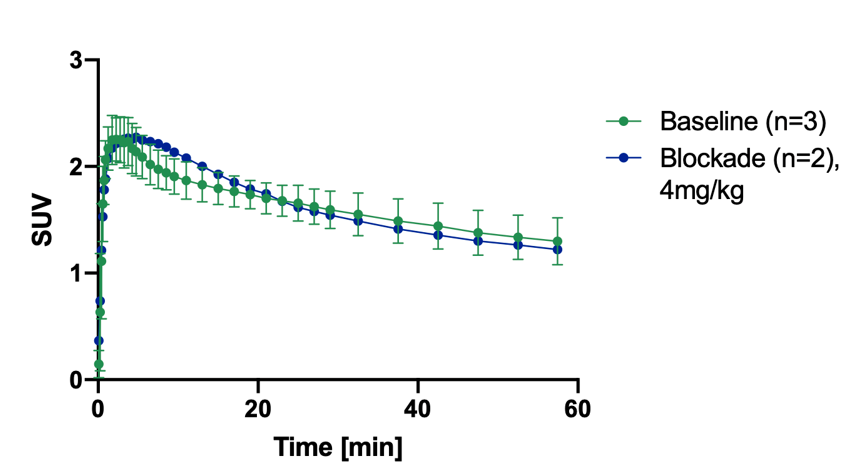


**Supplemental Figure 4**: Time-activity curves of the brain following administration of [^18^F]TAAR1-2203 to CD1 mice. Blockade conditions included the co-administration of non-radioactive TAAR1 agonist (RO5425754) at a dose of 4 mg/kg.

***Ex vivo* metabolite studies**


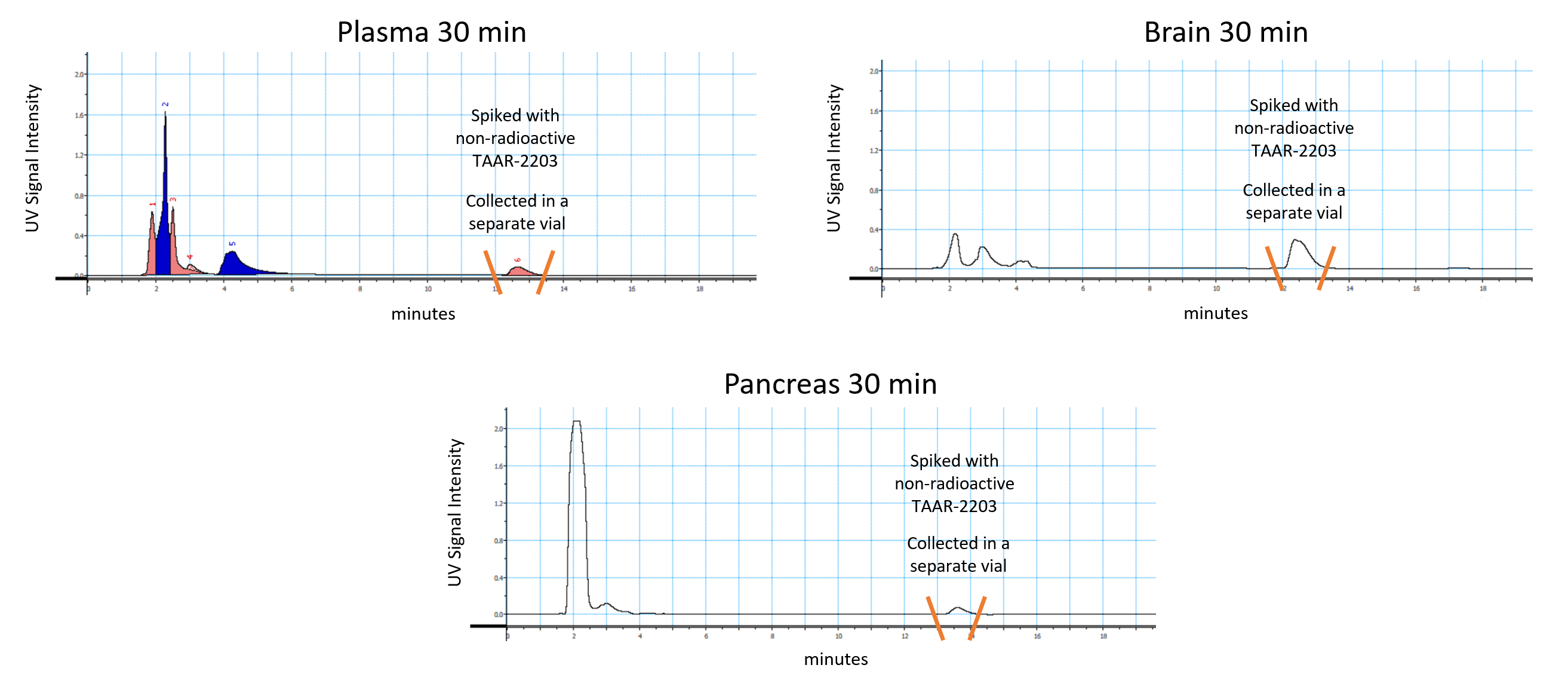


**Supplemental Figure 5**: UV chromatograms of *ex vivo* extracts from mouse plasma, brain, and pancreas at 30 minutes post-injection of [^18^F]TAAR1-2203. Each biological extract was spiked with a small amount of non-radioactive TAAR1-2203 immediately before HPLC analysis to enable visualization of the parent peak via UV detection. The UV signal depicted here was used to guide manual collection of the parent fraction (highlighted by orange markers), which was separated from the rest of the chromatogram. All collected fractions were then measured in a gamma counter to quantify radioactivity. This hybrid UV-guided fractionation approach ensured accurate assignment and quantification of intact parent compound versus radiometabolites, despite limited intrinsic radioactive signal in *ex vivo* samples.
